# Acute limb ischemia caused by ruptured cardiac hydatid cyst – A case report

**DOI:** 10.1016/j.ijscr.2018.12.005

**Published:** 2019-01-14

**Authors:** Samer Makki Mohamed Al-Hakkak, Firas Shaker Mahmoud Al-Faham, Ali Najeh Al-Awwady

**Affiliations:** a Department of Surgery, Faculty of Medicine, Jabir Ibn Hayyan Medical University, Najaf City, Iraq; b Department of Surgery, Faculty of Medicine, University of Karbala, Karbala City, Iraq

**Keywords:** Cardiac, Hydatid cyst, Limb ischemia, Left ventricle, Rupture, Embolectomy

## Abstract

**Introduction:**

Acute limb ischemia is a sudden decrease in limb perfusion that threatens the viability of the limb. Complete or even partial occlusion of the arterial supply to a limb can lead to rapid ischemia and poor functional outcomes within hours. In human echinococcosis cardiac involvement is a rare presentation, it may lead to life-threatening complications including cyst rupture; anaphylactic shock; tamponade; pulmonary, cerebral or peripheral arterial embolism. Cardiac hydatid cyst (CHC) may have different presentation include acute lower limb ischemia secondary to embolization from a ruptured cyst.

**Case presentation:**

We report an 18-year old male healthy building worker while he was working who presented with sudden onset acute right lower limb pain and paresthesia caused by rupture of primary CHC which managed as a surgical emergency.

**Discussion:**

Clinical presentation of ruptured of CHC depends on the specific location of the ruptured cyst that interferes and mobilization of daughter cyst that logged in vascular system with the function of the surrounding cardiac structures like our case that present with embolization of daughter cyst into the right external iliac artery which leads to acute limb ischemia.

**Conclusion:**

Cardiac hydatid cyst is a rare finding with a wide range of signs and symptoms. We are reporting this case to underline that cardiac hydatidosis should be considered as a differential diagnosis in young patients who suddenly develop acute limb ischaemic without a history of both cardiac diseases and trauma that lives in endemic regions of hydatidosis.

## Highlights

• Acute lower limb ischaemia in non-traumatic young age without history of cardiac disease should be consider rupture cardiac hydatid in mind.
• Echocardiography, computerized tomography play important role in diagnosis and early treatment.
• The diagnosis of hydatid cyst of the heart is difficult because of clinical and also radiographic findings may be nonspecific.
• Ruptured cardiac hydatid cyst should be diagnosed immediately and excision of the cardiac cyst should be performed as quickly as possible.

## 1 Introduction

Hydatid cyst disease is a significant health problem for undeveloped and developing countries. Human infestation occurs as an intermediary carrier by ingestion of the parasite’s eggs contaminated food. The parasite embryo gains access to systemic circulation through the intestine and it can reach any organ with different prevalence [1]. Cardiac hydatid cyst (CHC) is an infrequent type of involvement. It occurs in about 0.5–2% of cases, in comparison to the liver (65%) and the lung (25%). Therefore, (CHC) is a rare but potentially fatal site of pathology [2]. The larvae reach the heart through the coronary circulation in most of the cases. Additionally, though less infrequently, cardiac involvement can occur through the intestinal lymphatics, the thoracic duct, and the superior and inferior vena cava. Hemorrhoidal veins and the pulmonary veins may also be additional pathways [3,4]. Echinococcosis does not appear to be any age limit at presentation; it may manifest even in early childhood [[5], [6], [7], [8]]. It may be accompanied by multiorgan involvement [5,9]. The diagnosis of cardiac hydatid disease is based on a combination of clinical suspicion and cardiac imaging. Echocardiography and cardiac computerized tomography are highly sensitive and specific in the diagnosis of hydatid cysts [10].

## 2 Case presentation

**Fig. 1:**
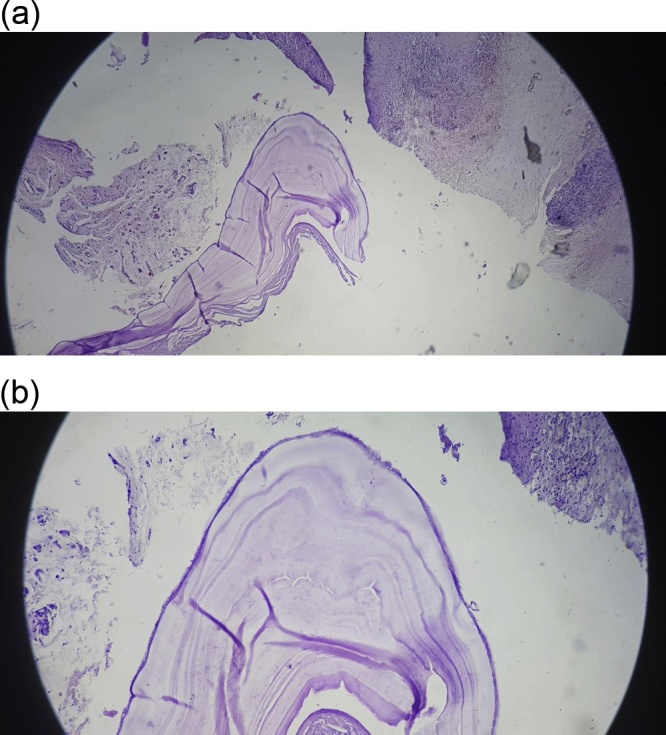
(a) Show laminated membrane which is typical for hydatid cyst. (b) Laminated membrane which is typical for hydatid.

**Fig. 2:**
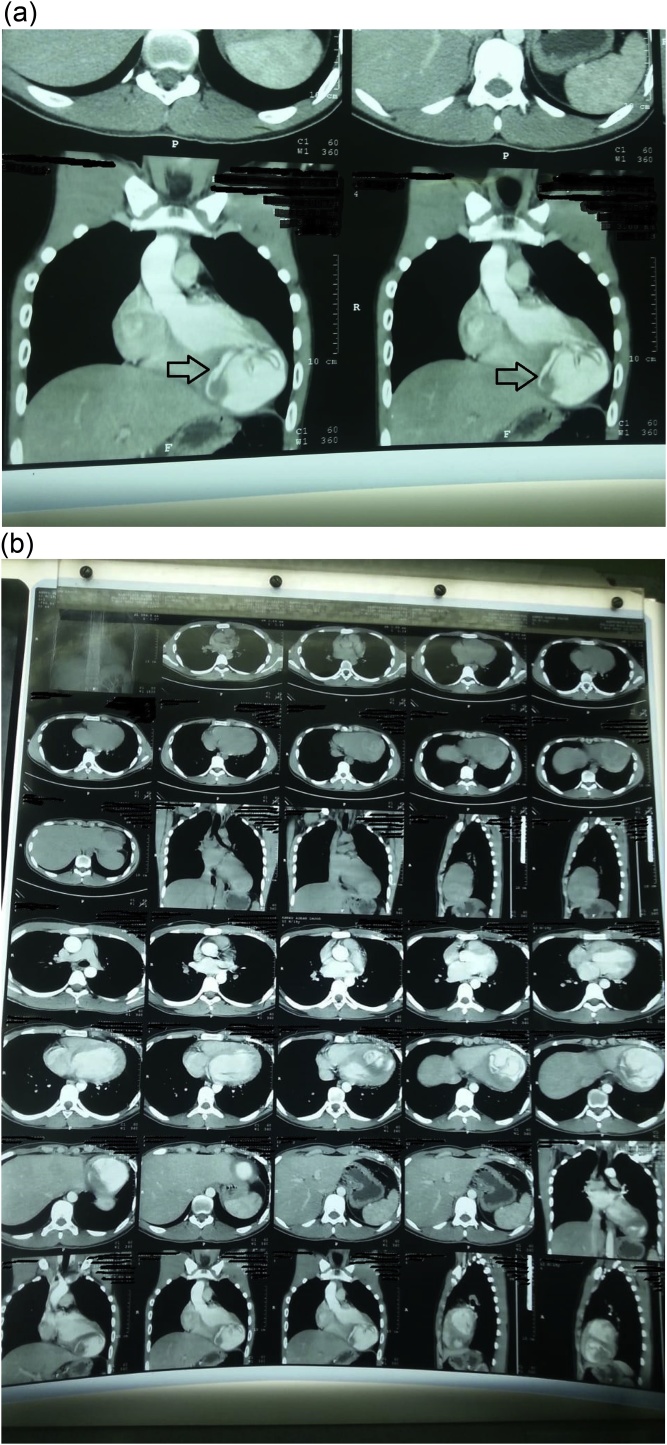

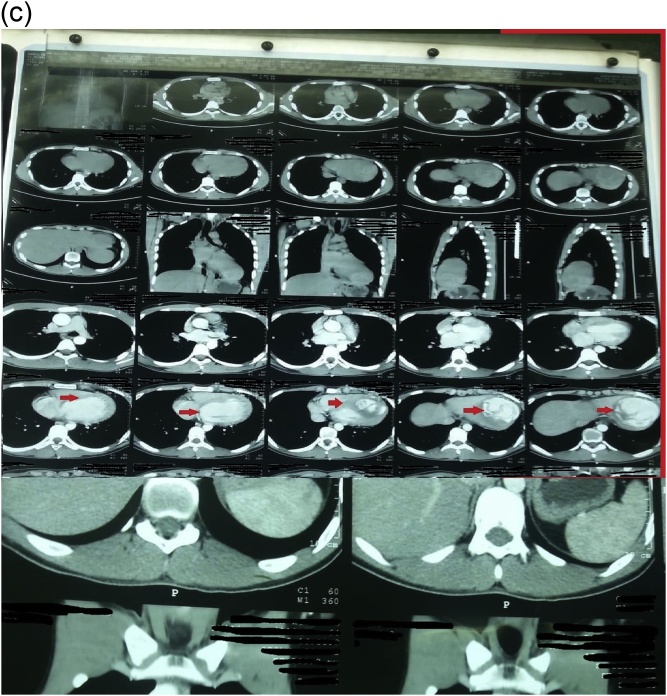
(a) Chest CT scan with hydatid cyst in the left ventricle (2). (b) CT scan of chest show left ventricle hydatid (2). (c) CT scan hydatid cyst in LV with membrane disloged and rupture (2).

**Fig. 3:**
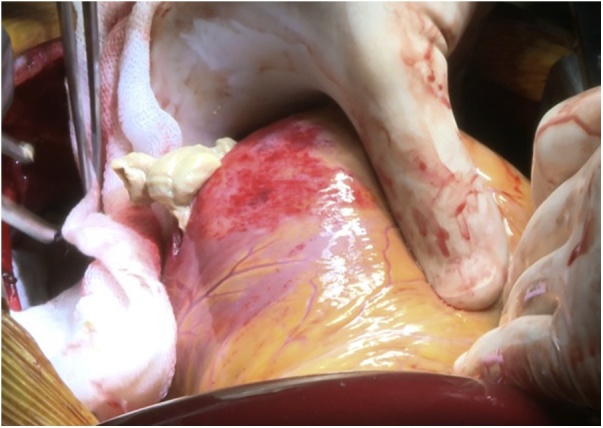
Left ventricular opening.

**Fig. 4:**
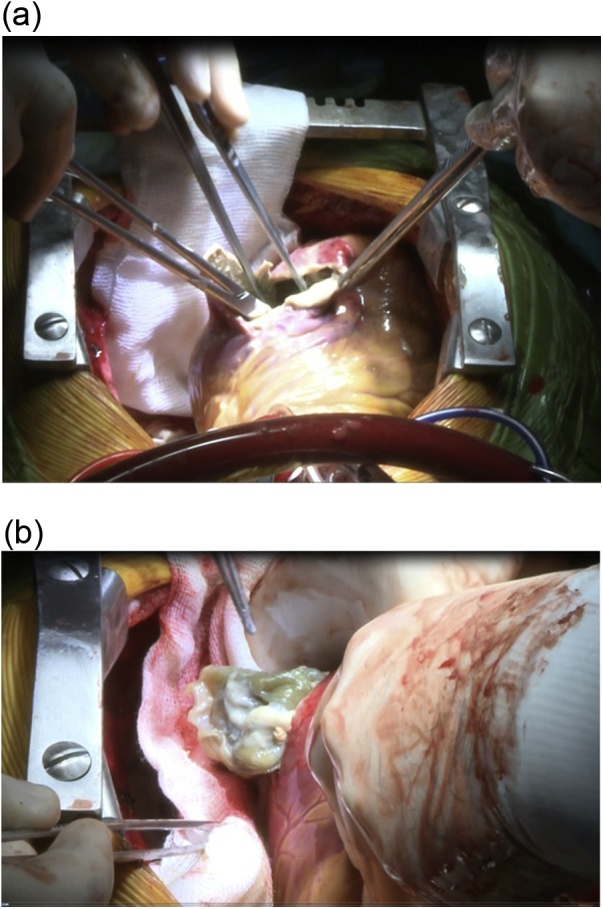
(a) Cleaning of endocyst. (b) Cyst excision from left ventricle.

**Fig. 5:**
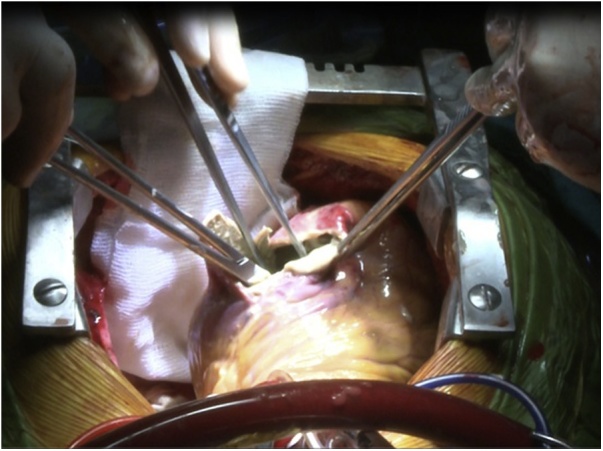
Mopping of endocyst well inside left ventricle.

**Fig. 6:**
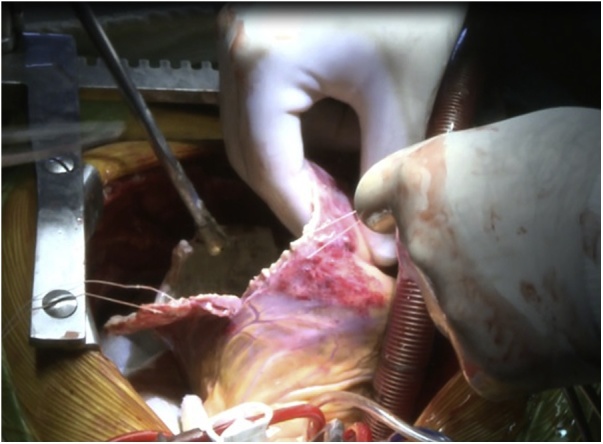
Stay suture and enlarge the cavity.

**Fig. 7:**
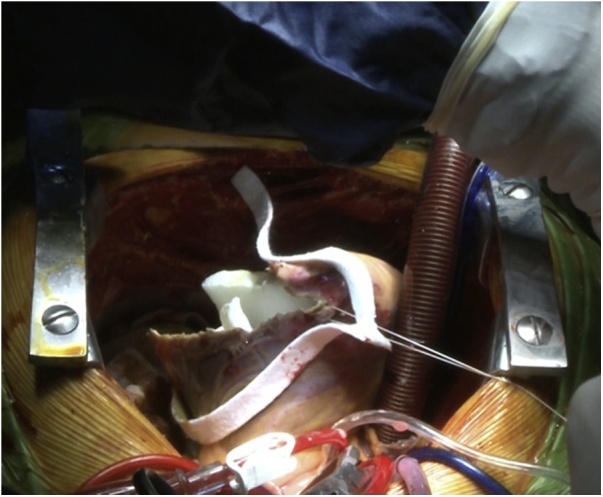
Putting the gelform inside the ectocyst.

**Fig. 8:**
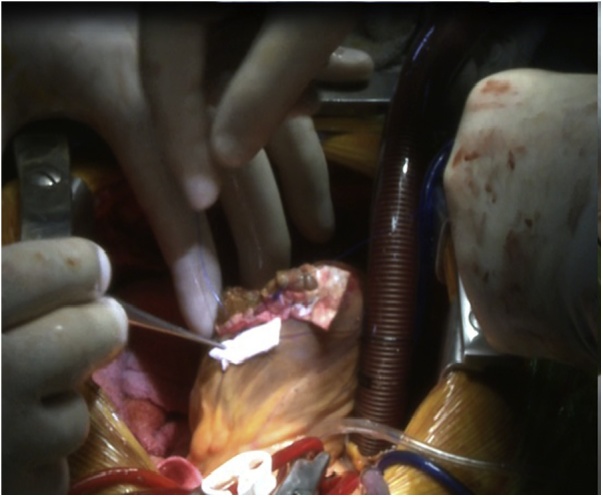
Closing the left venticle by matress suture.

An 18-year-old male worker was admitted to the emergency department due to pain, paresthesia, and coldness of the right lower limb. He has no history of cardiac disease or trauma and otherwise in good health. On physical examination, the right lower limb was cold and with cyanotic toes. The right femoral and popliteal pulses were undetectable. An urgent color-flow duplex scanning had been arranged which revealed a complete obstruction of the right external iliac artery blood flow by a rather big embolus. A trans-thoracic echocardiography showed an anechoic lesion of 36–40 mm originating from the left ventricle (LV). Since it was an endemic area of hydatid disease, a provisional diagnosis of (CHC) had been postulated. An urgent surgical embolectomy through the common femoral artery had been done. The obstruction has been opened with a Fogarty catheter. The catheter harvested a cruor thrombus with a white membrane from the common femoral artery, the histopathology of which was a hydatid tissue as it is shown in Fig. 1a and b. After immediate subsidence of symptoms and signs, and an uneventful post-operative night, a CT scan of thorax revealed a well-defined cystic lesion of 45 mm diameter was protruding into the LV, as shown in Fig. 2a, b, and c. A week later, the patient had been submitted to a standard a sternotomy and under cardiopulmonary bypass between the ascending aorta and the two-vena cava. The LV cavity showed an inside protruding mass, Fig. 3. The mass was incised and the cyst was removed, as shown in Fig. 4a and b). This has been followed by mopping of endocyst, as in Fig. 5, putting stay suture of LV as in Fig. 6, and enlarging of a cystic cavity as in Fig. 7. The perforated myocardium was sutured by prolene, and the cavity closure was achieved by a standard mattress suturing with gel foam, Fig. 8. The postoperative period was uneventful, and the patient was discharged after 9 days with no complications. The follow-up plan consisted of a standard albendazole treatment for 3 cycles each one for 28 days with a period of one-week rest to avoid drug complication. An abdominal ultrasound, and a single brain CT scan to rule out another organ involvement, all of which were negative.

## 3 Discussion

Cardiac Hydatid cysts of the left ventricle are usually located subepicardial and rarely rupture into the pericardial space. But in the right ventricle localization is subendocardial; rupture is more frequent and intracavitary rupture causes pulmonary embolization [[11], [12], [13]]. The literature states that the left ventricle CHCs are usually seen in the subepicardial space, they rarely rupture into the pericardial space. Right ventricle localization, in the other hand, is often subendocardial, with a higher chance of rupture and subsequent pulmonary embolization if it occurs intracavitary. The diagnosis of the left ventricular wall CHC is often difficult since its clinical and radiographic findings may be nonspecific. Patients with CHC may remain asymptomatic for many years or have minor nonspecific complaints, but it is associated with an increased risk of lethal complications if left undiagnosed and untreated [14]. Embolism of the external iliac artery by an Echinococcus cyst is extremely rare and is usually due to rupture of an intracardiac hydatid cyst. Most patients with CHC also have multiorgan involvement, while in our case, he had none. Only CHC present without any other organ involvement. CHC symptoms depend on the size and location of the cyst. Rupture into left-sided chambers may cause systemic emboli. Embolization to the lower limb artery is very rare [15,16]. CHC rupture has been presented with ischemia of femoral and popliteal. [17,18] To the best of our knowledge, this is the first reported case of right external iliac artery embolism in an adolescent patient due to ruptured CHC.

The duplex ultrasound, echocardiography, and computerized tomography play the important role in the diagnosis and early treatment of CHC, thus preventing life-threatening complications. Early recognition and prompt addressing of these complications particularly in the acute form of external iliac embolization, could be life-saving. Ruptured CHC should be suspected in young patients who have acute limb ischemia which comes from sheep-raising areas and/or if they have a suspected embolectomy material resembling germinative membrane [18].

The treatment for a CHC is urgently surgical, and no place for conservative approaches due to its morbidity-mortality potential. The diagnosed case should be provided with the standard medical treatment (Albendazole) and must be scheduled for further regular ultrasound examination for the whole follow-up period to anticipate any of future systemic occurrences or multi-organ involvement.

## 4 Conclusion

The peripheral arterial embolism is a rare manifestation of left ventricle CHC. Embolectomy and surgical resection of the cyst must be performed on an emergency basis. Patients must then undergo treatment by albendazole to prevent dissemination of the disease. In cases of vascular embolism developing suddenly in the extremities. It might be lifesaving to consider ruptured cardiac hydatid cyst in the differential diagnosis especially without a history of trauma particularly in endemic regions. The management of any organ hydatid cyst disease should rule out any possible cardiac involvement.

## Conflicts of interest

No any conflicts of interest.

## Sources of funding

None.

## Ethical approval

The study is exempt from ethical approval in my institution.

## Consent

Written informed consent was obtained from the patient for publication of this case report and accompanying images. A copy of the written consent is available for review by the Editor-in-Chief of this journal on request.

## Author contribution

I am the contributory author for this my case report. My colleague Dr Firas Al-Faham cardiothoracic surgeon and Dr Ali Al-Awwady assist me in contribute the case report.

## Registration of research studies

Research registry 4508.

## Guarantor

Samer Makki Al-Hakkak.

## Provenance and peer review

Not commissioned, externally peer-reviewed.

## References

[1] Dursun M., Terzibasioglu E., Yilmaz R., Cekrezi B., Olgar S., Nisli K. (2008). Cardiac hydatid disease: CT and MRI findings. AJR Am. J. Roentgenol..

[2] Tuncer E., Tas S.G., Mataraci I., Tuncer A., Donmez A.A., Aksut M., Yakut C. (2010). Surgical treatment of cardiac hydatid disease in 13 patients. Tex. Heart Inst. J..

[3] Niarchos C., Kounis G.N., Frangides C.R. (2007). Large hydatic cyst of the left ventricle associated with syncopal attacks. Int. J. Cardiol..

[4] Kammoun S., Frikha I., Fourati K. (2000). Hydatid cyst of the heart located in the interventricular septum. Can. J. Cardiol..

[5] Uysalel A., Aral A., Atalay S., Akalin H. (1996). Cardiac echinococcosis with multivisceral involvement. Pediatr. Cardiol..

[6] Emirogullari N., Uzum K., Ustunbas H.B., Andac H., Tasdemir K. (1995). Primary cardiac echinococcosis in childhood. Case report. Scand. J. Thorac. Cardiovasc. Surg..

[7] Turgut M., Benli K., Eryilmaz M. (1997). Secondary multiple intracranial hydatid cysts caused by intracerebral embolism of cardiac echinococcosis: an exceptional case of hydatidosis. Case report. J. Neurosurg..

[8] Akcakaya N., Soylemez Y., Cokugras H., Aytac A., Akalin F. (1994). A case of hydatid cyst with intramural cardiac localization. Scand. J. Infect. Dis..

[9] Atilgan D., Demirel S., Akkaya V., Korkut F. (1996). Left ventricular hydatid cyst: an unusual location of Echinococcus granulosus with multiple organ involvement. J. Am. Soc. Echocardiogr..

[10] Canpolat U., Yorgun H., Sunman H., Aytemir K. (2011). Cardiac hydatid cyst mimicking left ventricular aneurysm and diagnosed by magnetic resonance imaging. Turk Kardiyol. Dern. Ars..

[11] Bayezid O., Ocal A., Isik O., Okay T., Yakut C. (1991). A case of cardiac hydatid cyst localized on the interventricular septum and causing pulmonary emboli. J. Cardiovasc. Surg. (Torino).

[12] Ege E., Soysal O., Gulculer M., Ozdemir H., Pac M. (1997). Cardiac hydatid cyst causing massive pulmonary embolism. Thorac. Cardiovasc. Surg..

[13] Pasaoglu I., Dogan R., Hizan E., Oram A., Bozer A.Y. (1992). Right ventricular hydatid cyst causing recurrent pulmonary emboli. Eur. J. Cardiothorac. Surg..

[14] Madariaga I., de la Fuente A., Lezaun R., Imizcoz M.A., Carmona J.R., Urquia M. (1984). Cardiac echinococcosis and systemic embolism: report of a case. Thorac. Cardiovasc. Surg..

[15] Di Bello R., Menendez H. (1963). Intracardiac rupture of hydatid cysts of the heart. A study based on 3 personal observations and 101 cases in the world literature. Circulation.

[16] Perez-Gomez F., Duran H., Tamamer S., Pervote L., Blanes A. (1973). Cardiac echinococcosis: clinical pictures and complications. Br. Heart J..

[17] Öztürk Mehmet, Sığırcı Ahmet, Dağlı1 AdileFerda (2015). A rare cause of embolism in the popliteal artery of an adolescent: ruptured cardiac hydatid cyst. Anatol. J. Cardiol..

[18] Hèla B.J., Abir B., Majdi G., Aiman D., Iheb S., Nizar E., Sayda M., Imed F. (2015). Interventricular septum hydatid cyst presenting with acute lower limb ischemia: a case report. Libyan J. Med..

